# Correlation between Oxidative Stress, Nutrition, and Cancer Initiation

**DOI:** 10.3390/ijms18071544

**Published:** 2017-07-17

**Authors:** Subbroto Kumar Saha, Soo Bin Lee, Jihye Won, Hye Yeon Choi, Kyeongseok Kim, Gwang-Mo Yang, Ahmed Abdal Dayem, Ssang-goo Cho

**Affiliations:** Department of Stem Cell and Regenerative Biotechnology, Incurable Disease Animal Model & Stem Cell Institute (IDASI), Konkuk University, Seoul 05029, Korea; subbroto@konkuk.ac.kr (S.K.S.); soobineey@naver.com (S.B.L.); wjh021@naver.com (J.W.); hyeon.choi24@gmail.com (H.Y.C.); proproggs@naver.com (K.K.); slayersgod@nate.com (G.-M.Y.); ahmed_morsy86@yahoo.com (A.A.D.)

**Keywords:** nutrition, oxidative stress, reactive oxygen species, cancer progression

## Abstract

Inadequate or excessive nutrient consumption leads to oxidative stress, which may disrupt oxidative homeostasis, activate a cascade of molecular pathways, and alter the metabolic status of various tissues. Several foods and consumption patterns have been associated with various cancers and approximately 30–35% of the cancer cases are correlated with overnutrition or malnutrition. However, several contradictory studies are available regarding the association between diet and cancer risk, which remains to be elucidated. Concurrently, oxidative stress is a crucial factor for cancer progression and therapy. Nutritional oxidative stress may be induced by an imbalance between antioxidant defense and pro-oxidant load due to inadequate or excess nutrient supply. Oxidative stress is a physiological state where high levels of reactive oxygen species (ROS) and free radicals are generated. Several signaling pathways associated with carcinogenesis can additionally control ROS generation and regulate ROS downstream mechanisms, which could have potential implications in anticancer research. Cancer initiation may be modulated by the nutrition-mediated elevation in ROS levels, which can stimulate cancer initiation by triggering DNA mutations, damage, and pro-oncogenic signaling. Therefore, in this review, we have provided an overview of the relationship between nutrition, oxidative stress, and cancer initiation, and evaluated the impact of nutrient-mediated regulation of antioxidant capability against cancer therapy.

## 1. Introduction

Nutrition is proposed to play an essential role in cancer progression. Cancer is the second leading cause of deaths in people from developed countries, whereas it is the most leading cause of death in people from developing or underdeveloped countries [1,2]. According to the International Agency for Research on Cancer (IARC), more than 10 million new cases and about 10 million fatal cases have occurred due to cancer onset worldwide [3]. In western countries, more than 65% of all the cancers occur upon exposure to numerous harmful substances, such as those present in western-style diet, alcohol, and smoking, that do not exist naturally in the environment [4].

Nutrition can also cause oxidative stress, augment a cascade of molecular reactions in cells, and alter the metabolic state of tissues [5]. Oxidative metabolism and redox homeostasis are suggested to be an essential part of aerobic life [6]. Living organisms cannot survive without these processes. Under such unfavorable conditions, oxygen derivatives can damage nucleic acids, lipids, and proteins; alter oxidative equilibrium; and regulate cell viability [7]. Oxidative stress induces the formation of excess antioxidants to protect the human body from antioxidant deficiency [8]. Moreover, nutrition can induce oxidative stress even in normal physiological conditions in the human body, and dietary factors can also serve as inflammatory and pro-oxidant factors [9]. Thus, nutritional oxidative stress might be described as a postprandial imbalance between the antioxidant defense and the pro-oxidant load as a consequence of inadequate or excess supply of nutrients [10].

Oxidative stress is known as a physiological state in which high levels of reactive oxygen species (ROS) and free radicals are generated due to antioxidant metabolism [11]. Normal cellular metabolism produces ROS and free radicals and plays a crucial role in cell signaling pathways [12]. Mechanically, mitochondria, the largest powerhouse of cells, generate ROS when generating adenosine triphosphate (ATP), whereby electrons react with oxygen (O_2_) and subsequently form the superoxide anion (O_2_^−^) [13]. There are several studies confirming that oxidative stress may have a core relationship with human pathophysiological diseases [14,15,16]. Specifically, oxidative stress is prominently known to damage the DNA molecule, alter signaling pathways, and regulate progression of various cancers, including those of the breast, lung, liver, colon, prostate, ovary, and brain [17,18,19,20,21,22,23]. Moreover, it is reported that the whole DNA molecule can bind with hydroxyl radicals, and consequently, damage the deoxyribose backbone, including purine and pyrimidine bases. During these damaging processes, 8-OH deoxyguanosine (8-OHdG) can be produced, which may markedly increase the risk of mutagenesis [24]. The 8-OHdG molecules are also used as indicators to detect free radicals during DNA mutagenesis and are widely implicated as an early detection tool for cancer progression [24,25]. Importantly, 8-OHdG can transform GC pairs to TA pairs upon DNA replication, which might induce mutagenesis if oxidative lesions exist, subsequently causing cancer initiation [26].

The precise mechanisms underlying induction of oxidative stress by nutrition followed by cancer initiation are the current research topics, and the probable mechanisms include alterations in epigenetic events and induction of genomic instability, which alter gene expression, cause resistance to apoptosis, and induce tumor invasion and metastasis [12,14,15,16,24,26]. Therefore, in this review, we have provided an overview of the correlation between nutrition, oxidative stress, and carcinogenesis.

## 2. Correlation between Nutrition and Oxidative Stress

It is known that overnutrition may generate free radicals, and subsequently elevate oxidative stress [27] and ROS-mediated modulation of various molecular pathways [28,29,30]; therefore, scientists have directed increasing attention towards investigation of the function of oxidative stress in various pathophysiological diseases and normal body metabolism [16,31,32,33,34,35]. Therefore, the antioxidant capability of the human body is considered a crucial factor for overcoming free radical-mediated oxidative stress and the subsequent pathophysiological processes.

### 2.1. Nutrition Induces Oxidative Stress during Early Human Development

There are various crucial environmental factors, including nutrition, involved in epigenetic modifications [36]. For instance, undernutrition or malnutrition and low birth weight in utero due to early infant growth deficiency may be closely linked to risk factors, such as insulin resistance, obesity, reproductive dysregulation, and cardiovascular disease, in adulthood [37,38]. Similarly, offspring grown in a prenatally rich nutritional circumstance is at an increased risk of compromised fertility and cardiometabolic disorders later in life [39,40]. A recent study proposed that oxidative stress has a potent effect in nutrition-mediated epigenetic changes in various experimental models [41].

Obesity, maternal malnutrition, or obesogenic maternal diet upon gestation, but not in the post-weaning period [42], is associated with augmented oxidative stress markers and diminished antioxidant capability in the offspring, resulting in diabetogenic effects [43,44]. Concurrently, antioxidant supplementation could significantly attenuate obesity in their offspring [45]. Nutrition may trigger epigenetic changes in perinatal development into adulthood via different pathways, such as metabolic risk factor progression and oxidative stress generation.

### 2.2. Nutrition Triggers Oxidative Stress at the Cellular Level

A previous study demonstrated that after glucose intake, mononuclear (MNC) and polymorphonuclear (PMN) leukocytes of normal subjects generate ROS and induce inflammation due to excess micronutrients [46]. Similarly, after lipid intake, leukocytes in normal subjects may also significantly induce ROS generation and inflammation; protein intake can trigger ROS generation, but to a much lesser degree than glucose and lipid intake can [47]. Moreover, upon assessing a mixed meal in well-fit subjects, severe inflammatory alterations were identified, with a reduction in inhibitor κBα (IκBα), and upregulation of binding of nuclear factor κB (NF-κB) and expression of inhibitory proteins p47^phox^ subunit, IκB kinase α (IKKα), IκB kinase β (IKKβ), and plasma C-reactive protein (CRP) [48].

Postprandial oxidative stress might increase due to excessive caloric intake, which abnormally increases blood glucose, free fatty acids (FFA), and triglycerides circulating in the blood. These high concentrations of FFA and glucose outpace the entire capability of mitochondria for oxidative phosphorylation, ultimately leading to improved transfer from single electrons to molecular oxygen; consequently, O_2_^−^ enters the circulation [49,50]. Besides mitochondria, ROS production by leukocytes is also induced by the caloric amount, as previous studies indicated that caloric limit led to a decent reduction in ROS production via lipid peroxidation and protein carboxylation [51,52,53].

Inappropriate lifestyle patterns of an individual, including physical inactivity or obesity, can also cause ROS production in the postprandial state. As a result, obese individuals experience pernicious and acute oxidative stress after a fatty meal, compared to responses of the non-obese well-fitted individuals [54]. Inconsistent data exist regarding the outcome of exercise in postprandial oxidative stress. Although exercise is thought as a tool to increase endogenous antioxidant defenses, numerous researchers have been unsuccessful in showing a positive effect of physical activity on postprandial oxidative stress [55,56,57].

Cooking method can also have a postprandial impact on oxidative metabolism. Protein- and fat-rich food cooked quickly under high temperatures lead to the formation of dietary advanced glycation end products (AGEs) [58]. Studies showed that a single oral challenge by AGEs (coke) caused severe postprandial endothelial dysfunction, as illustrated by a significant reduction in flow-mediated dilatation both in diabetic and in healthy subjects [59]. Nutritive AGEs appear to affect reproductively challenged women as well. A study in women with polycystic ovarian syndrome (PCOS) showed that low-AGE meals in combination with six-month treatment with orlistat (a lipase inhibitor) led to a significant improvement of their hormonal profile and body mass index (BMI) [60].

**Figure 1:**
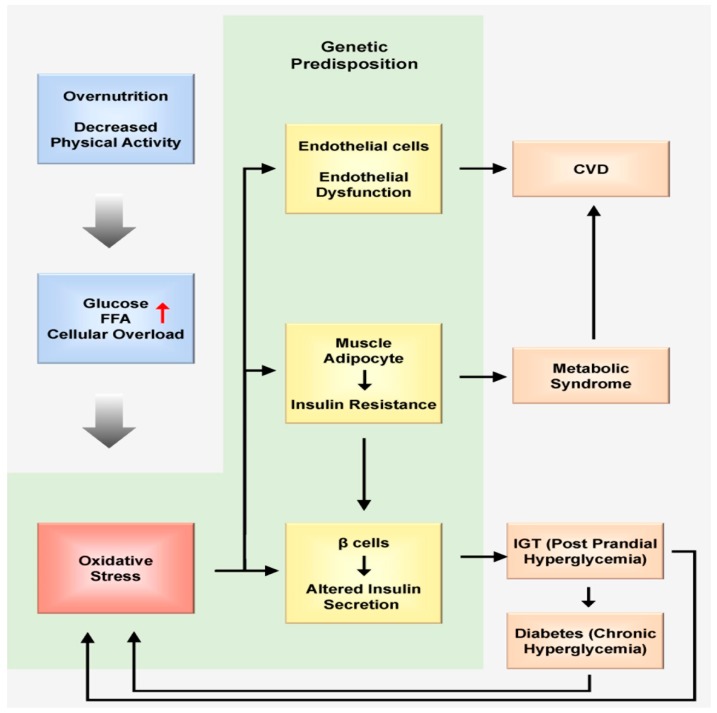
Overnutrition and decreased physical activity lead to overloaded glucose and free fatty acid (FFA) levels in cells. Their conversion into energy is supplemented by augmented free radical generation (oxidative stress). The muscle adipocytes can defend themselves from this situation and exhibit insulin resistance, aiming to decrease glucose and FFA permeation into the cells. The endothelial and β cells are insulin-independent. In these cells, glucose and FFA overload may cause oxidative stress, which in turn induces dysfunction of both endothelial and β cells. Endothelial dysfunction may induce cardiovascular disease (CVD), and β cell dysfunction is characterized by altered insulin secretion. β cell dysfunction is particularly characterized by a decrease in first-phase insulin secretion, which in turn produces the clinical situation of impaired glucose tolerance (IGT). This last condition is clinically characterized by increased postprandial hyperglycemia. Postprandial hyperglycemia induces oxidative stress. The persistence of this condition exhausts β cells, leading to overt diabetes. Oxidative stress produced during both IGT and overt diabetes may contribute to the development of CVD. Moreover, the cluster of risk factors that accompany insulin resistance also contributes to CVD development. Red colored arrow represents overload (Adapted from [49]).

Taken together, increasing evidence demonstrates that nutrition triggers major oxidative and inflammatory imbalances in the postprandial state. Indeed, postprandial hyperlipidemia and hyperglycemia, or so-called postprandial dysfunction in the body, are gradually gaining vital consideration as major risk factors for some diseases. Continuous accumulation of all these imbalances during the constant postprandial state that symbolizes current lifestyles may contribute to the pathophysiology of reproductive and metabolic disorders (Figure 1).

### 2.3. Nutrition Increases Oxidative Stress during Tissue Metabolism

**Figure 2:**
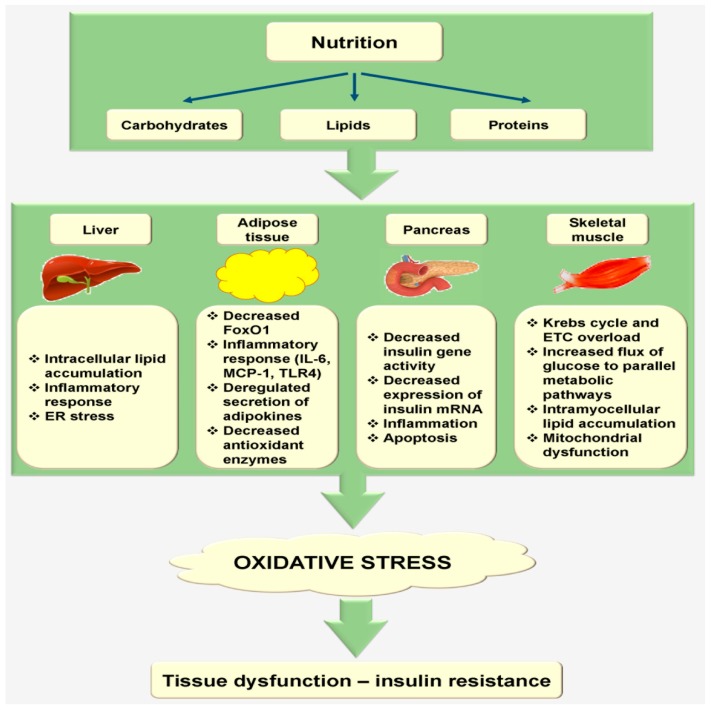
Nutrition mediates oxidative stress at the metabolic tissue level. Dietary fat (lipids) induces intracellular lipid accumulation in the liver and subsequently causes the inflammatory response and ER stress, which ultimately results in oxidative stress- and insulin resistance-induced liver dysfunction. A nutritious diet can induce the inflammatory response and impair FoxO1 expression, adipokine secretions, and antioxidant enzyme activity in the adipose tissue, resulting in an increased ROS generation, which ultimately causes dysfunction of the adipose tissue. In pancreatic β-cells, hyperglycemia can induce mitochondrial ROS production promoting a native oxidative microenvironment, which unfortunately changes insulin gene expression and activity that further increases oxidative stress, including inflammation generation, consequently collapsing β-cell function. Overfeeding and increased dietary fat (lipids) appeared to enhance mitochondrial dysfunction, with decreased ATP synthesis, attenuated mitochondrial gene expression, and augmented ROS generation. Consequently, a vicious cycle occurs as these mitochondrial dysfunctions further intensify the metabolic abnormalities of the skeletal muscle. ER: endoplasmic reticulum, FoxO1: Forkhead box protein O1, IL-6: Interleukin 6, MCP-1: Monocyte chemoattractant protein-1, TLR4: Toll-like receptor 4, ETC: electron transport chain. (Adapted from [60]).

Nutrient consumption elicits a major oxidative and inflammatory effect at the cellular level, which alters tissue metabolism. Nutritional oxidative stress after carbohydrate, protein, and lipid intake results in a domino of metabolic alterations in various tissues, including the liver, adipose tissue, pancreatic β-cells, and skeletal muscle. These active but metabolically distressed tissues interacting with nutrients further augment oxidative stress, eventually resulting in an infinite vicious cycle (Figure 2).

#### 2.3.1. Liver

Dietary fat intake or overfeeding augments free fatty acid (FFA) supply in the liver, which can affect liver metabolism by the accumulation of intracellular lipids. In the liver tissue, increased malonyl-CoA levels stimulate de novo FA production and prevent carnitine palmitoyltransferase-1 (CPT-1) function. Consequently, fatty acids (FAs) cannot be broken down in the mitochondria and are diverted to other metabolic pathways, resulting in the formation of ceramides, diacylglycerol (DAG), and triacylglycerol (TAG) [61]. In a rat model, fat-rich meal administration for only three days led to a three-fold increase in liver lipid accumulation, without any significant growth in the skeletal muscle or visceral fat content, suggesting that liver insulin resistance may precede systemic insulin resistance (Figure 2) [62]. As stated above, these lipids recruit numerous inflammatory factors that derestrict insulin signaling, including the c-Jun N-terminal kinase (JNK) and protein kinase C (PKC) pathways. Additionally, in an investigational model, FFA-containing cultured hepatocytes exhibited augmented levels of prothrombotic and oxidative markers, such as nitric oxide (NO), plasminogen activator inhibitor-1 (PAI-1), and malondialdehyde (MDA) [63]. Concurrently, massive substrate supply and liver overfeeding expose the ER to a substantial anabolic load that accordingly stimulates ER stress and protein misfolding, which can induce inflammatory signaling activation and ROS generation (Figure 2) [61]. Lastly, lipid accumulation in the hepatic cells affects hepatic glucose production in impaired insulin-mediated suppression and hyperlipidemia, categorized by elevated hepatic clearance of high-density lipoprotein (HDL)-cholesterol combined with elevated secretion of very low-density lipoproteins (VLDL) [64].

#### 2.3.2. Adipose Tissue

In the adipose tissue, ROS production and oxidative metabolism play major roles in adipogenesis [65]. Various sources are involved in producing intracellular ROS in adipocytes. Although adipocytes are not thought to be pure energy-producing cells, ROS may be generated from electron transport chain (ETC) substrate overload as well as from mitochondria [66]. Moreover, several enzymes can induce ROS generation in adipocytes, including nicotinamide adenine dinucleotide phosphate (NADPH) oxidase. In adipocytes, NADPH oxidase 4 (NOX4) is the core isoform and its expression is augmented in the fat cells upon exposure to enriched nutrient derivatives, including glucose or palmitate [67]. Knockdown of NOX4 in adipocytes (3T3-L1 cells) prevented glucose- and palmitate-stimulated ROS production, indicating the significance of non-mitochondrial ROS in adipocytes [68].

Upon intake of a meal, an inflammatory response occurs in the adipose tissue [69]. A study conducted on rat visceral adipose tissue showed that rats fed with a fatty meal showed an acute postprandial stimulation of inflammatory signaling [70]. Similarly, in humans, 6 h after the feeding of a mixed meal, a similar upregulation of MCP-1 and IL-6 was noted within the adipose tissue in normal-weight, overweight, and obese subjects, independent of the grade of adiposity (Figure 2) [71]. In addition, the change in postprandial inflammatory effects in the adipose tissue due to the specific quantity and quality of dietary fat was studied by various scientific groups, but their results are conflicting. A study involving 75 subjects with metabolic syndrome revealed that as compared to long-term ingestion of saturated fat diet, that of high-monounsaturated fat diet led to a weakened postprandial inflammatory effect in the adipose tissue [72], whereas another study indicated that individuals with metabolic syndrome displayed impaired postprandial adipose tissue inflammation, regardless of the quantity and the quality of fat ingested [73]. From the direct stimulation of inflammatory pathways by nutrient consumption, a high-fat diet may prompt native inflammation in the adipose tissue through the discharge of unnecessary FFAs. The responses of FFAs in the inflammatory pathways are facilitated through the Toll-like receptor (TLR-4), which further induces the secretion of different cytokines and macrophage aggregation in the adipose tissue (Figure 2) [74].

Overall, oxidative stress can also be identified postprandially in adipocytes. In cultured adipocytes, elevated FFA levels augmented oxidative stress via NADPH oxidase stimulation, and oxidative stress directly caused dysfunctional secretion of adipokines. Additionally, increased ROS generation caused by increased expression of NADPH oxidase and decreased expression of antioxidative enzymes was investigated in the adipose tissue of overweight mice [75]. Thus, nutrition-activated oxidative stress likely leads to a contrary native redox status that could affect the role of free radicals in the adipose tissue (Figure 2) [76].

#### 2.3.3. Pancreas

Oxidative stress can also likely compromise pancreatic β-cell function, as β-cells are inherently sensitive to oxidative stress. In a previous study, β-cells exposed to H_2_O_2_ generated cyclin- and p21-dependent kinase inhibitors and downregulated insulin mRNA, calcium flux, and ATP reduction in the cytosol and mitochondria [77]. Moreover, β-cells express low levels of antioxidant enzymes, such as catalase, superoxide dismutase (SOD), and glutathione peroxidase, and are more sensitive to detrimental ROS actions [78]. Hence, oxidative stress, induced by elevated FFA and glucose levels, insulin resistance, and long-term inflammation through the above-stated mechanisms, clearly plays a role in pancreatic cells and alters insulin secretion (Figure 2) [16].

In patients with diabetes, long-term induction of plasma FFA and glucose levels has damaging effects on the pancreatic cell function [16]. An in vitro study showed that the islets or HIT-T15 cells cultured in high concentrations of FFA and glucose exhibited reduced levels of insulin mRNA and gene function and altered glucose-induced insulin secretion pathway [79]. Aberrant free radical production and oxidative stress could be one of the crucial mechanisms underlying these instabilities (Figure 2). Moreover, hyperglycemia by itself can augment intracellular mitochondrial ROS generation in pancreatic β-cells, triggering a native oxidative microenvironment, which incidentally alters several metabolic signaling pathways that further intensify oxidative stress [80], including long-term low-grade AGE and inflammation generation, consequently collapsing β-cell function (Figure 2) [81].

#### 2.3.4. Skeletal Muscle

Regarding metabolic circulation, the skeletal muscle can also be characterized as a pathway controller. This tissue represents a crucial source of energy generation and accounts for approximately 80% of the postprandial insulin-induced glucose dumping [82]. As a pure energy-generating organ, skeletal muscle is packed with mitochondria that control energy homeostasis.

After nutrient feeding, insulin induces glucose entry in the skeletal muscle through glucose transporter type 4 (GLUT4) [83]. This is a cardinal phase in the body’s metabolic pathways as fuel consumption should be attuned to fuel obtainability. The capability of skeletal muscle to mainly shift from lipid oxidation and high amounts of FA utilization in fasting situations to glucose ingestion, oxidation, and storage under insulin-prompted circumstances is recognized as metabolic flexibility. The inability to shift from lipid to carbohydrate use (metabolic inflexibility) was investigated in obese patients and is accompanied with intra-myocellular lipid aggregation and insulin resistance (Figure 2) [84]. Numerous factors regulate the metabolic flexibility of a subject, including nutrient presence, plasma FFA levels, the accessibility of the adipose tissue for lipid storage, and their level of physical activity [85]. Another factor that may be associated with metabolic flexibility is mitochondrial oxidative capability. Although a study showed contradictory data, it was suggested that mitochondrial aberrations in the muscle could stimulate metabolic flexibility to lipids and prompt insulin resistance (Figure 2) [85].

In the skeletal muscle, dietary habits may also disturb physiological metabolic developments and their role through direct changes in the mitochondrial biology [86]. Together, increased dietary fat and overfeeding appeared to induce mitochondrial inactivity, with declined ATP synthesis, altered mitochondrial gene expression, and augmented ROS generation. Consequently, a vicious cycle occurs as these mitochondrial dysfunctions further intensify the metabolic abnormalities of the skeletal muscle (Figure 2).

### 2.4. Nutrition Induces Oxidative Homeostasis

Nutrition-stimulated inflammatory and oxidative status in severe settings can alter extracellular and intracellular physiological activities. When these instabilities are recurrent, they execute a persistent inflammatory and oxidative response, which, in some cases, can prompt multiple diseases.

Limited-calorie dietary patterns can provoke the precise reverse effect, promoting cell longevity and securing oxidative balance. For instance, six months of caloric limitation significantly diminished oxidative stress and declined fasting insulin levels and body core temperature in healthy subjects [87]. Moreover, the study showed improved basal endothelial function and augmented plasma antioxidant capability in patients with diabetes, who followed a Mediterranean diet for three months in comparison with those patients on control diets [88].

Overall, evidence suggests that diet regulates oxidative stability both in an acute and in a chronic state. Nutritional variance can easily interrupt this cellular stability, initiate unfavorable pathophysiological pathways, and stimulate the incidence of numerous diseases in humans.

## 3. The Relationship between Nutrition and Oxidative Stress Following Carcinogenesis

**Figure 3:**
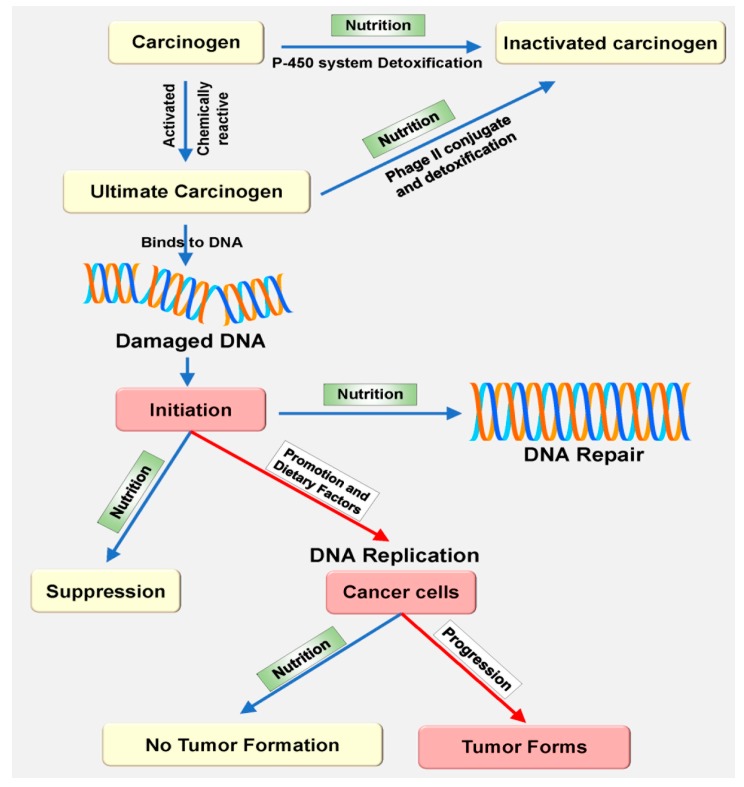
Nutrition as a mediator of cancer suppression at the molecular level. A chemically reactive and activated form of pro-carcinogen or carcinogen (ultimate carcinogen) is capable of direct covalent binding to protein and/or nucleic acid macromolecules. It directly binds to a cell component (probably DNA) to initiate carcinogenesis. The preventive function of nutrition can be activated by the enzymes (cytochrome P450) in carcinogenesis. Cancer cells can form a tumor by the action of various dietary factors. Metabolically active nutritional compounds can defend carcinogenesis by suppressing the activity of carcinogen or by inducing DNA repair mechanism. Blue colored arrows represent beneficial effect and red colored arrows represent harmful effect of nutrition [99,100,101,102].

**Figure 4:**
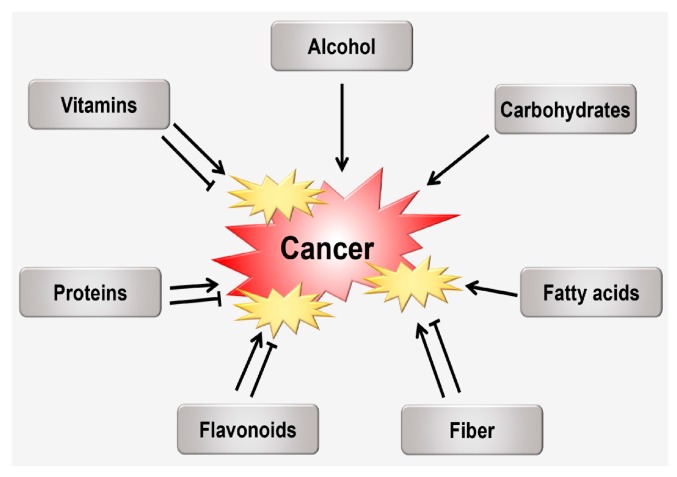
Some vital dietary factors have been associated with various aspects of cancer progression. Arrows represent activation of cancer and T bar represent inhibition.

The worldwide cancer burden is anticipated to increase by more than two-fold over the next two decades [89], therefore worsening a massive public health and medical care problem. Physical activity, nutrition, and diet rank high among the most important risk factors for human cancer, in part because of their influences on obesity, which is a recognized risk factor for various malignancies [90,91,92,93,94,95]. The role of some specific nutrients in cancer etiology has been proposed based on associations stated in epidemiological studies, further supported by biological credibility. The ultimate carcinogen is known as chemically reactive and activated form of a pro-carcinogen or carcinogen that is capable of a direct covalent binding to protein and/or nucleic acid macromolecules. The ultimate carcinogen directly binds with a cell component (probably DNA) to initiate carcinogenesis. These factors are linked to the antioxidant status of selected nutrients, impact on epigenetic functions, DNA adducts, DNA repair, regulation of gene expression, inflammation, stimulation of growth factors, or influence on circulating intensities of endogenous hormones (Figure 3) [96,97,98]. Incessant exposure to environmental carcinogens and inhalation chemicals is assumed to induce the amount of cytochrome P450 CYP1A1 expression in extrahepatic tissues via the aryl hydrocarbon receptor (AhR) [99,100,101,102]. Though the latter has long been identified as a ligand-activated transcription factor (TF), which is accountable for the xenobiotic inducing pathway of numerous phase I and phase II metabolizing enzymes, recent studies propose that AhR is associated with several cell signaling pathways critical to cell cycle modulation and normal homeostasis [101,102]. Alteration of these pathways is associated with tumor progression. Moreover, it is increasingly evident that P450 plays a vital role in the detoxification of environmental carcinogens, following the metabolic activation of dietary compounds (nutrition) with cancer preventative activity (Figure 3) [102]. Along with other crucial factors, such as diet, energy balance, BMI, physical activity, and metabolic rate, nutrition may also influence DNA replication of cancer cells following cancer progression. Therefore, nutrition-mediated oxidative stress plays a crucial role in carcinogenesis. Some of the vital dietary components that have an association with oxidative stress following different aspects of carcinogenesis have been discussed in this section (Table 1 and Figure 4).

### 3.1. Alcohol

Alcohol is a prominent carcinogen linked with breast, oropharyngeal, colorectal, liver, and esophageal cancers [103]. Excessive consumption of alcohol also leads to fibrotic changes in the liver [104,105]. Moreover, it leads to the production of ROS following oxidative stress, which, consequently, causes severe dysfunction and damage to the biological signaling molecules [106]. Additionally, it disrupts intra- and extra-cellular network and functions, which ultimately cause chromosomal abnormalities, DNA damage, DNA methylation modification, signaling pathway alteration, tumor necrosis factor α (TNF-α) release, and retinoid metabolism impairment, consequently, leading to cancer initiation [107,108,109,110]. Functional diversity in the genes associated with alcohol metabolism can result in varying exposure to the carcinogenic metabolites of alcohol; therefore, identifying genetic intolerance to alcohol can aid in cancer prognosis [111]. For instance, people with a common genetic mutation in the alcohol dehydrogenase gene that suppresses enzyme activity have a higher risk of esophageal cancer than those who have a fully active enzyme [103]. Alcohol facilitates its mutagenic effects by the derivation of acetaldehyde adducts, induction of the activity of Kupffer cells, and enhancing oxidative stress by augmenting formation of gut-derived endotoxins [110]. Alcoholism results in accumulation of acetaldehyde, which, consequently, causes genotoxicity. A similar change occurs due to accumulated acetaldehyde in hepatocellular carcinoma [112,113]. Moreover, according to World Cancer Research Fund (WCRF) analysis, alcohol intake is significantly correlated with increased breast cancer risk [90]. Numerous epidemiological studies supported a positive interaction between breast cancer risk and alcohol [114]. A meta-analysis revealed that high alcohol consumption (10 g of ethanol consumption per day) was highly associated with risks for ER^+^PR^+^, ER^+^PR^−^, ER^+^, and ER^−^ breast tumors, but not ER^−^PR^−^ tumors [115]. Additionally, there are several contradictory studies on the probable relationship of alcohol consumption with numerous histological grades or stages of prostate cancer [116,117,118,119,120]. Previous meta-analyses have also emphasized these irregularities, highlighting the necessity for further studies in this area [121,122].

### 3.2. Carbohydrates

Ingestion of nutritional carbohydrate, a key dietary factor, disturbs an individual’s glycemic response and insulin secretion, while consequences differ depending on the amount of carbohydrates consumed [167]. Carbohydrate quality could affect cancer risk, especially, that of breast cancer, significantly by influencing plasma levels of glucose and insulin, and insulin resistance [130]. Recent meta-analysis studies described a potential relationship between glycemic index (GI), degree of cancer risk, and intake of carbohydrate quality [168,169,170,171]. Previous studies suggest that oxidative stress may have an important role connecting acute hyperglycemia to augmented cardiovascular risk [172,173,174]. Acute enhancement in blood glucose concentrations may increase the formation of free radicals by an imbalance in the ratio of NADH to NAD and by non-enzymatic glycation increased by glucose in cells [175,176]. The direct indication from studies presented that enhanced hyperglycemia or meal consumption and its derived glucose can promote oxidative stress and impair antioxidant defenses [177,178]. Consequently, oxidative stress was significantly augmented after food intake that produced a superior degree of hyperglycemia in both normal subjects and those with diabetes [179]. According to the European Prospective Investigation into Cancer and Nutrition (EPIC), increased carbohydrate and glycemic burden in the food were associated with an increase in ER^−^/PR^−^ and ER^−^ breast cancer among older women [180]. Similarly, the Women’s Health Initiative (WHI) suggested that consuming foods with high insulinogenic content may increase the risk of breast cancer [131]. Together, the potential relationship between cancer risk and dietary GI was more commonly stated by case-controls than by the cohort studies. A probable purpose for this is that case-control reports are more liable to problems of remembering and selection difficulty than cohort studies are. In addition, most case-control studies were conducted in Europe and most cohort studies were conducted in North America. The diverse results between studies performed in North Americans and Europeans may also reveal variances in nutritional lifestyles between the two regions. Individuals from Europe ingest carbohydrate-enriched food and different kinds of carbohydrates [181] compared to individuals in North America [182], who consistently consume more fats. Studies are often unable to demonstrate a relationship between oxidative stress-induced cancer risk and carbohydrate intake.

### 3.3. Fatty acids (FAs)

Dietary lipids or fats are frequently blamed as the key source of superfluous energy. When caloric consumption surpasses energy expenses, the resultant substrate-induced enhancement in citric acid cycle activity produces an excess of ROS. Moreover, dietary FA ingestion influences the relative FA configuration of biological membranes defining its sensibility to oxidative changes [183]. There are huge controversies around finding a relationship between FA-rich meals and cancer risk in population-based reports, despite a solid biological credibility underlying these relationships. The role of inflammation in membrane fluidity and functions, stimulation of growth factors, and regulation of gene expression, or its effect on circulating levels of endogenous hormones has been cited. Recent data demonstrate a link between dietary FA with induced oxidative stress and carcinogenesis in the rat model [184]. Several epidemiological studies mention that, rather than total dietary fat ingestion, subgroups of FAs could differentially affect cancer risk [185,186,187,188]. Essential FAs (EFAs) of the omega-3 family (α-linolenic acid, docosahexaenoic acid (DHA), and eicosapentaenoic acid (EPA)) and omega-6 family (arachidonic acid and linoleic acid) have been a vast subject of study, because of their dietary significance and their association with the prognosis of various types of cancers. In spite of numerous studies conducted over the last decades, recent scientific data are debatable and there is a lack of reliable conclusions about the effect of EFAs and the risk of breast, bladder, colorectal, lung, or prostate cancers [189,190,191,192]. In the broad literature regarding this type of EFA (omega-3, omega-6, and omega-3/omega-6 ratio) and its relationship to cancer progression, several underlying mechanisms have been hypothesized. One of the most established mechanisms is an association between inflammatory pathways and the function of omega-3 and omega-6 FAs on the action of cyclooxygenase-2 (COX-2) in prostate cancer [134,135,136]. On the contrary, Gao et al. [193] demonstrated that palmitate, a saturated FA, up-regulated COX-2 via NF-κB-dependent mechanism; consequently, COX-2-associated oxidative stress weakened endothelium-dependent relaxations in the mouse aortas. However, metabolic characteristics of these EFAs are completely conflicting. The COX-2 enzyme can convert omega-6 FAs into prostaglandin E2, a pro-inflammatory cytokine, which enables angiogenesis and cell proliferation, whereas prostaglandin E3 is produced from omega-3 FAs with the help of COX-2, which does not facilitate mitogenic characteristics [194].

This proposal could elucidate the results achieved by assessing the impact of the omega-3/omega-6 ratio on melanoma [195], and the effects of DHA- and EPA-rich fish oil on colorectal [196] or prostate cancer, where the diversity of results leads to contradictory conclusions [197,198].

### 3.4. Fiber

Consumption of whole grain cereals, vegetables, and fruits provides the fibers necessary for our health, with the recommended intake being approximately 21–38 g/day. The protective action of fibers is not only associated with colorectal cancer, but also with other cancer types. A study showed an 11% decrease in breast cancer risk in individuals consuming a fiber-rich diet versus that in individuals consuming the lowest amount of fiber [142]. This association is dose-dependent; cancer risk decreased 7% with each 10 g/day of fiber intake, which is not dependent on the ethnic group, region, or menopausal status [142]. Moreover, the WCRF assessment board concluded an inadequate level of data regarding the relationship between dietary fiber and breast cancer risk [90]. Similarly, an organized review and meta-analysis of potential studies presented a significant inverse relationship between nutritional fiber intake and breast cancer risk [143]. In addition, the recent epidemiological proof is not convincing regarding the ability of fiber intake to decrease colorectal cancer risk. Some studies have shown significant results, with up to a 25% reduction in cancer risk by ingesting around 12.6–33.1 g/day of fiber, or 17% reduction by consuming fiber three times a day, though some studies have not found any beneficial effects [144,199].

### 3.5. Flavonoids

Cancer initiation and progression have been associated with oxidative stress by enhancing DNA mutations or increasing DNA damage, genome variability, and cell proliferation, and hence antioxidant agents could intervene with carcinogenesis [200]. Among the antioxidant compounds, isoflavones are the most well-known compounds that possess well-characterized anti-estrogenic activity (antagonistic for the β-estrogen receptor); functions in intracellular steroid metabolism (inhibiting the enzyme that transforms androgen to estrogen); and anti-angiogenic, anti-proliferative, and pro-apoptotic activities in various tumor cells [149,150,151]. Other flavonoid compounds, polyphenols, have anticancer activity both in humans and animal models [201,202]. Currently, increasing attention is directed towards the role of natural antioxidant agents on modulating intracellular ROS levels resulting into epigenetic alterations of essential genes in tumorigenesis [202]. Several flavonoids were confirmed to disrupt the enzymes leading to epigenetic modifications, which regulate the inflammation process that might oscillate in cancer [202]. Excessive ROS generation may lead to tissue injury that may induce inflammatory process [203], the inflammatory mediators may be involved in various chronic diseases, including CVD, neurological disease, and carcinogenesis [204]. Although in vitro studies depict a positive outcome, case-control results and phase III clinical trials afford unconvincing data for certain kinds of tumors, such as breast or prostate neoplasms [151,205]. A study on Asian women revealed that isoflavone consumption of 20 mg/day can decrease breast cancer risk by 29% as compared to that after consumption of 5 mg/day [152]. On the contrary, according to a meta-analysis, no association was found in western women, even though these women ingested 0.8 mg of isoflavones per day [151]. Previously, studies have stated that Asian men consume high amounts of isoflavone-containing foods, while western counterparts consume mostly red meat-containing foods with minimal isoflavones [206,207,208]. This variation in results can be caused by numerous factors, including dose and type of isoflavones, type of cancer, or even diverse enzymatic polymorphisms between subjects [209].

### 3.6. Proteins

In a nutritional diet, protein is the most important element for human health. Proteins contain no nutritional value until they are digested by protease and peptidase enzymes. Excessive protein consumption can induce amino acid oxidation and urea synthesis [210], and impair the nutritional efficacy of energy utilization [211]. An interesting study stated that high protein intake could obliterate the stability of antioxidants and oxidation of amino acids in the digestive system of mice and promote generation of ROS in the digestive gland [212]. A conceivable explanation is that ROS might be generated after meat consumption during its metabolism [213]. Moreover, high-protein ingestion can result in oxidative stress, inducing risk for long-term diseases, including carcinogenesis [214,215,216]. In patients with cancer, protein consumption is decreased tremendously due to reduced digestion, low food intake, and augmented catabolism [217]. Recently, an epidemiological study showed that intake of protein-rich food (especially animal protein) could be associated with a higher risk of cancer [157]. Moreover, a few epidemiological studies have discovered an association between intake of animal protein (e.g., red meats) and several diseases (e.g., hypertension and colon cancer) [218,219]. There are no particular enduring clinical trials analyzing meatless diets for children or adults. Similarly, there is little evidence indicating that colorectal cancer progression occurs upon satisfactory consumption of animal protein [158]. Recent studies from large cohorts, such as the Health Professional Follow-up Study, the Nurse’s Health Study, and the Multiethnic Cohort, depicted insignificant or inverse correlations between ingestion of unrefined red meat and colon cancer [218,220]. Together, research from the interference studies on cancer and diet, including the Polyp Prevention Trial and the Women’s Health Initiative, found that a reduction in dietary consumption of animal protein (e.g., processed meat and red meat) did not decrease the risk of colon cancer and/or had no outcome on adenoma relapse in the large bowel [221,222,223].

### 3.7. Vitamins

Recent epidemiological studies have been conducted to discover the association between vitamin consumption and the risk of cancer diagnosis. According to previous studies, numerous vitamins, including vitamin A, B, C, D, and E, have been implicated in the risk of cancer occurrence [161,162,163,164,165]. Vitamins C, D, and E and selenium share fundamental antioxidant properties and all protect against oxidative stress and its harmful effects in our body that lead to carcinogenesis. However, oxidative stress is a natural process with positive outcomes, such as improved immune response [224]. Previous studies stated that high-dose vitamin C killed cancer cells by playing a role as a pro-drug, which provides hydrogen peroxide (H_2_O_2_) [225,226,227]. Vitamin C-induced elevated levels of ROS, including H_2_O_2_, are considered to play a vital role in carcinogenesis [226]. Previous studies also reported that vitamin C administration promoted cytotoxicity by ATP reduction in some cancer cells [227,228,229]. A case-control study involving women from Klang Valley and Selangor, Malaysia, demonstrated that a good antioxidant consumption, including vitamins A and E, can reduce oxidative stress and subsequently prevent breast cancer risk [230]. The relationships between breast cancer and B vitamins have been broadly studied and these relationships are complex. From questionnaires, epidemiological studies have estimated an association between folate consumption and the risk of breast cancer with conflicting results [231]. On the contrary, preventive effects have been witnessed in individuals with low folate consumption and occasional vitamin intake [232]. Moreover, there are questionable findings for vitamin B in prostate cancer [233], for vitamins C and E in liver [234] and prostate cancers [235], and for folic acid and vitamin D in pancreatic cancer [236,237].

## 4. The Association between Oxidative Stress and Cancer Progression

An association between oxidative stress and cellular alteration was first recognized in 1981 when it was identified that insulin raised intracellular H_2_O_2_ levels and augmented tumor cell proliferation [238]. After more than three decades, the function of ROS in cancer progression remains conflicting. Oxidative stress is involved in various diseases, including neurodegenerative diseases [239,240], chronic inflammation [241,242], metabolic disorders [243,244], and extensively in various cancers [245,246,247,248,249]. The rise in ROS levels from oxidative stress, as a consequence of oncogene signaling pathways, may exploit underlying mutagenesis and genomic variability in cancer cells to stimulate cancer progression. Cancer cells require high levels of ATP because it acts as “fuel” for aberrant cell proliferation. However, the effect of this excess energy generation is the accumulation of ROS, which needs to be prevented by scavenging actions to ensure cell survival [250]. To prevent these possibly toxic effects of ROS, numerous oncogenes also augment the expression of nuclear factor erythroid 2-related factor 2 (NRF2), which diminishes ROS levels and stimulates tumorigenesis [251]. Similarly, NRF2 not only offers protection against chemical carcinogens, but also augments cancer progression by defending cancer cells from ROS and DNA damage [252,253,254,255,256,257,258]. In contrast, NRF2 deletion in pancreatic cancer cells augmented DNA damage and inhibited carcinogenesis [251].

**Figure 5:**
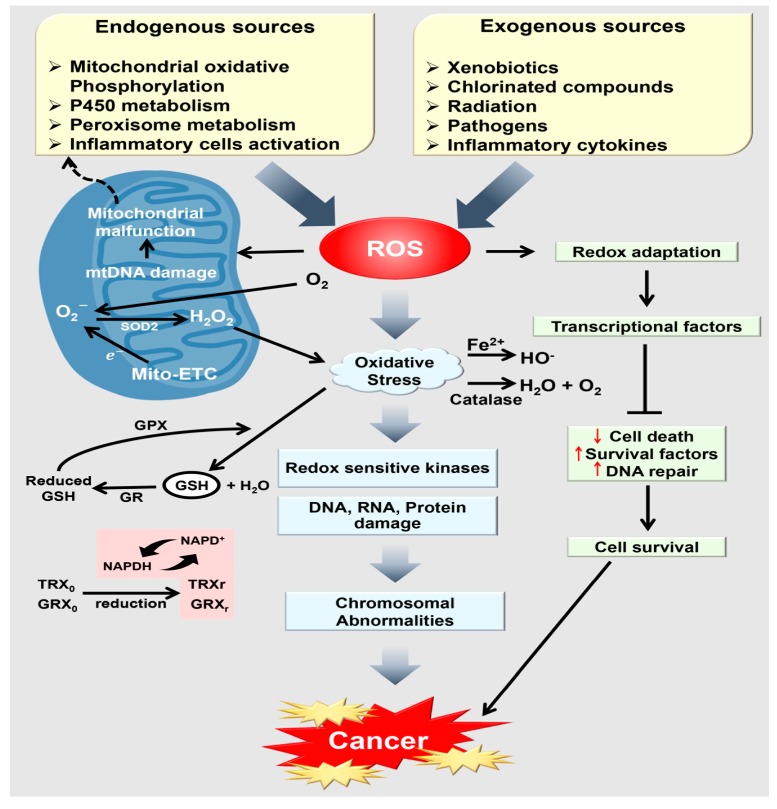
A schematic diagram of overall signaling pathways of cancer progression induced by oxidative stress. SOD: superoxide dismutases; Mito-ETC: mitochondrial electron transport chain, GSH: glutathione; GR: glutathione reductase; GPX: glutathione peroxidase; GRXo, glutaredoxin (oxidized); GRXr: glutaredoxin (reduced); GSHr: glutathione (reduced); TRXo, thioredoxin (oxidized); TRXr: thioredoxin (reduced). Black arrows represent activation and T bar represent inhibition, red colored arrows represent upregulation/downregulation. (Adapted from [273]).

Several studies have assessed ROS levels and generation under numerous conditions with the aim of determining when ROS are carcinogenic and when they are cancer suppressive [259]. At low or endurable levels, ROS may aid cancer progression either by playing as signaling elements or by stimulating alterations in genomic DNA or DNA damage. For example, ROS can promote expression of cyclin D1, phosphorylation of extracellular signal-regulated kinase (ERK) and JUN N-terminal kinase (JNK), and activation of mitogen-activated protein kinase (MAPK), all of which are connected to cancer progression and survival [260,261,262,263,264,265]. Moreover, ROS have been found to inversely incapacitate tumor suppressors, including protein tyrosine phosphatases (PTPs) and phosphatase and tensin homolog (PTEN), due to the existence of the redox-sensitive cysteine residues that exist in their catalytic sites [266,267,268]. Remarkably, PTPs can also control signaling pathways to induce the expression of antioxidant enzymes and diminish ROS levels [269]. Additionally, normal stem cell renewal and differentiation are controlled by ROS levels [270]; while cancer stem cells (CSCs) share similar properties with normal stem cells, comparatively little is known regarding their association with redox status. Recently, studies have shown that the liver and breast cancer stem cells tend to have low ROS levels, leading to the augmented expression of ROS-scavenging signaling proteins [270]. If CSC growth is vital for tumor initiation, then retaining low ROS levels in CSCs may be essential for the endurance of pre-neoplastic foci. Hence, although chemotherapy and radiotherapy prompt ROS generation, they are beneficial for abolishing most cancer cells, yet may be unable to cure the patient, leading to the greater capability of CSCs to endure in circumstances of high ROS by increasing antioxidants levels [250]. As ROS are debatable mediators of the adverse effects of some anticancer drugs and ionizing radiation, CSCs may be favorably released and aggressively selected by actions that depend on increased ROS levels. Furthermore, the supplementary oxidative stress prompted by these actions may cause further mutations and DNA damage, resulting in the expansion of drug-resistant cancer cells (Figure 5).

At elevated levels, ROS stimulate cell death and harmful cellular damage. In this case, cancer cells must overcome increased levels of ROS, particularly at initial stages of cancer progression. A recent study found that circumstances that enhance oxidative stress also raise the specific pressure on pre-neoplastic cells to induce influential antioxidant mechanisms [271]. Increased levels of ROS are also prompted by dissipation from the cell matrix [272]. This feature is relevant during metastasis of cancer cells that need to survive upon migration to distant organs. Thus, cancer cells typically have a high antioxidant capability that controls ROS levels and are attuned with biological functions of the cell, but are quite higher than the antioxidant capacity of normal cells. Moreover, increased ROS levels by endogenous antioxidants are unfavorable to cancer cells as well as cancer progression. We consider that targeting these enriched antioxidant protective mechanisms may represent an approach that can precisely destroy cancer cells, including CSCs, while sparing normal cells.

## 5. Conclusions

In the human body, nutrition is one of the vital regulators of oxidative stress. Nutrient consumption and the associated postprandial oxidative stress result in the accumulation of molecular alterations in the crucial signaling pathways of several organs, critically changing the cellular milieu. However, the particular pathophysiological roles of oxidative stress and nutrition are still elusive, with targeted therapeutic modalities representing a puzzling field. Specifically, when the organs of the gastrointestinal (GI) tract are exposed to the highest amount of dietary associated carcinogens, the injurious effects of these components affect the whole body system. Over the past decades, extensive studies have revealed that alterations in the cell metabolism play a vital role in the progression of various types of cancer. In general, carcinogenesis as well as dietary carcinogen-associated carcinogenesis, is significantly correlated with chronic and/or acute oxidative stress. The precise nature of the effect of oxidative stress on cancer development and/or response to treatment requires further exploration. The association between nutrition and oxidative stress may have an important role in cancer and CSC progression as well as therapy. To validate and confirm all of these above-mentioned hypotheses, more detailed further investigations and research are required. Recently developed technologies, including metabolomics and deep DNA sequencing, are imperative tools that would support to define how the metabolism of cancer cells become accustomed and offers a buffer against augmented oxidative stress. However, the pathophysiological relationship between carcinogenesis and oxidative stress opens prospects for protective and even therapeutic use of beneficial, healthy dietary compounds indicated as nutraceuticals. Therefore, this review details our understanding of the correlation between nutrition, oxidative stress, and cancer development, and uncovers related crucial therapeutic strategies.

## Figures and Tables

**Table 1:** The role of various dietary components in oxidative stress and carcinogenesis.

No.	Dietary Components	Role in Oxidative Stress	Role in Carcinogenesis
1	Alcohol	▪ Promotes ROS production while lowering cellular antioxidant levels, thereby altering homeostasis between pro- and anti-oxidants leading to oxidative stress in multiple tissues [123]. ▪ Increases ROS production and oxidative stress, and results in the accumulation of acetaldehyde [124]. ▪ alters mitochondrial function resulting in cellular death [125].	▪ Prominent carcinogen linked with several cancers [95]. ▪ Higher risk for esophageal cancer [95]. ▪ Highly associated with risks for breast tumors [115]. ▪ Alcohol intake and the genes involved in alcohol metabolism and their interaction increase the risk of breast cancer in post-menopausal women [126]. ▪ Chronic alcohol abuse can cause folate deficiency, which is a well-documented risk factor for breast cancer [127].
2	Carbohydrates	▪ Lead to increased oxidative stress, which has been associated with increased risk for atherosclerosis and related disorders [128]. ▪ High-carbohydrate meal may evoke a greater postprandial oxidative stress response [129].	▪ Could affect breast cancer influencing plasma levels of glucose and insulin, and insulin resistance [130]. ▪ Consuming foods with high insulinogenic content may increase the risk of breast cancer [131].
3	Fatty acids (FAs)	▪ Omega-3 FAs reduce oxidative stress [132]. ▪ FAs shorten in chain length and decrease unsaturation and peroxidation, while the 1-carbon cycle shifts from the methylation to the transsulfuration pathway [133].	▪ Established mechanism is an association between inflammatory pathways and the function of omega-3 and omega-6 FAs on the action of cyclooxygenase-2 (COX-2) in prostate cancer [134,135,136]. ▪ n-3 FAs, especially the long-chain polyunsaturated FAs, eicosapentaenoic acid and docosahexaenoic acid, present in fatty fish and fish oils inhibit carcinogenesis [137].
4	Fiber	▪ Could protect from oxidative stress [138]. ▪ Reduced levels of oxidative stress [139]. ▪ Elicited modest improvements in indices of oxidative stress and inflammation [140]. ▪ Dietary fiber supplementation, rather than energy intake and dietary restriction, appears to be the main process regarding oxidative stress in the cardiac tissue [141].	▪ An 11% decrease in breast cancer risk in individuals consuming a fiber-rich diet versus that in individuals consuming the lowest amount of fiber [142]. ▪ With up to a 25% reduction in cancer risk when ingesting around 12.6–33.1 g/day of fiber, or 17% reduction for consuming fiber 3 times a day [143,144]. ▪ It reduces the risk of developing some types of cancer [145].
5	Flavonoids	▪ Prevent disuse muscle atrophy by attenuating oxidative stress derived from mitochondrial dysfunction [146]. ▪ Have potential antioxidant actions by reacting with and inactivating O_2_^−^, oxygen lipid peroxide radicals, and/or stabilizing free radicals involved in the oxidative process by hydrogenation or complexing with oxidant species [147]. ▪ Have both a cytoprotective effect owing to ROS scavenging and cytotoxic effect caused by H_2_O_2_ generation [148].	▪ Isoflavones are the most well-known compounds that possess well-characterized anti-estrogenic activity; functions in intracellular steroid metabolism; and anti-angiogenic, anti-proliferative, and pro-apoptotic activities in various tumor cells [149,150,151]. ▪ Isoflavones consumption of 20 mg/day can decrease breast cancer risk by 29% compared to that by consumption of 5 mg/day [152]. ▪ Flavonoids are potent regulators of cyclin B and p21 required for cell cycle progression, which may play some roles in the prevention of carcinogenesis [153]. ▪ Flavonoids have emerged as potential chemopreventive candidates for cancer treatment, especially, by their ability to induce apoptosis [154].
6	Proteins	▪ Long-term intake of high protein diets did not increase variables of oxidative stress [155]. ▪ Become activated by oxidation and help bacteria to respond to oxidative stress [156].	▪ Protein-rich food (especially animal protein) could be associated with a higher risk of cancer [157]. ▪ Colorectal cancer progression occurs upon satisfactory consumption of animal protein [158].
7	Vitamins	▪ Vitamin A is rapidly oxidized in the presence of oxygen, transient metals, and light [159]. ▪ Vitamin E plays an important protective antioxidant role in elderly, particularly in conditions where oxidative stress and free radicals are potentiated [160].	▪ Numerous vitamins, including vitamin A, B, C, D, and E, have been implicated in the risk of cancer occurrence [161,162,163,164,165]. ▪ Intake or synthesis of vitamin D is associated with reduced incidence and death rates of colon, breast, prostate, and ovarian cancers [166].

## Abbreviations

8-OHdG	8-OH deoxyguanosine
AGE	advanced glycation end product
ATP	adenosine triphosphate
BMI	body mass index
COX-2	cyclooxygenase 2
CPT-1	carnitine palmitoyltransferase-1
CRP	C-reactive protein
CSC	cancer stem cells
CVD	cardiovascular disease
DAG	diacylglycerols
DHA	docosahexaenoic acid
EFA	Essential fatty acids
EPA	eicosapentaenoic acid
EPIC	European Prospective Investigation into Cancer and Nutrition
ER:	endoplasmic reticulum
ERK	extracellular signal-regulated kinase
ETC:	electron transport chain
FFA	free fatty acids
FoxO1	Forkhead box protein O1
GI	glycemic indexes
GLUT4	glucose transporter type 4
GPX	glutathione peroxidase
GR	glutathione reductase
GRXo	glutaredoxin (oxidized)
GRXr	glutaredoxin (reduced)
GSHr	glutathione (reduced)
HDL	high-density lipoproteins
IARC	International Agency for Research on Cancer
IGT	impaired glucose tolerance
IKKα	IκB kinase α
IKKβ	IκB kinase β
IL- 6	Interleukin 6
IκBα	inhibitor κBα
JNK	c-Jun N-terminal kinase
MAPK	mitogen-activated protein kinase
MCP-1	Monocyte chemoattractant protein-1
MDA	malondialdehyde
Mito-ETC	mitochondrial electron transport chain
MNC	mononuclear cells
NADPH	nicotinamide adenine dinucleotide phosphate
NF-κB	nuclear factor κB
NO	nitric oxide
NOX4	NADPH oxidase 4
NRF2	nuclear factor erythroid 2-related factor 2
PAI-1	plasminogen activator inhibitor-1
PCOS	polycystic ovarian syndrome
PKC	protein kinase C
PMNL	polymorphonuclear leukocytes
PTEN	phosphatase and tensin homolog
PTP	protein tyrosine phosphatase
ROS	reactive oxygen species
SOD	superoxide dismutase
TAG	triacylglycerol
TLR4:	Toll-like receptor 4
TRXo	thioredoxin (oxidized)
TRXr	thioredoxin (reduced)
VLDL	very low-density lipoproteins
WCRF	World Cancer Research Fund
WHI	Women’s Health Initiative
WHO	World Health Organization
